# CCL2 produced by pancreatic ductal adenocarcinoma is essential for the accumulation and activation of monocytic myeloid‐derived suppressor cells

**DOI:** 10.1002/iid3.523

**Published:** 2021-09-15

**Authors:** Haitao Gu, Wensheng Deng, Zhong Zheng, Ke Wu, Feng Sun

**Affiliations:** ^1^ Department of General Surgery, School of Medicine, Shanghai General Hospital Shanghai Jiao Tong University Shanghai China; ^2^ Department of Urology, School of Medicine, Renji Hospital Shanghai Jiao Tong University Shanghai China; ^3^ Department of Urology, School of Medicine, Shanghai General Hospital Shanghai Jiao Tong University Shanghai China

**Keywords:** CCL2, immune suppression, M‐MDSCs, pancreatic ductal adenocarcinoma

## Abstract

**Introduction:**

The development of pancreatic ductal adenocarcinoma (PDAC) is closely tied with the immune system. C‐C motif chemokine ligands (CCL) were proved to lead to immune recruitment and training. Thus, we reckoned CCL2 to be the kernel of immune suppression in PDAC tissues.

**Methods:**

We compared normal pancreatic tissues with PDAC tissues according to The Cancer Genome Atlas (TCGA) and clinical samples. Flow cytometry was used to identify M‐MDSCs. We further demonstrated immune suppression of M‐MDSCs according to proliferation rates of CD8^+^ T cells/CD4^+^ T cells. Levels of reactive oxygen species (ROS) and Arginase were also tested by flow cytometry, enzyme‐linked immunosorbent assay, and western blot analysis. We also analyzed the specific mechanisms by cluster analysis after CCL2 stimulating M‐MDSCs.

**Results:**

We found that CCL2 highly increased in PDAC tissues. CCL2 is positively related to CD33 and CD14, markers of monocytic myeloid‐derived suppressor cells (M‐MDSCs). We have demonstrated that CCL2 recruited M‐MDSCs into PDAC tissues both in vitro and in vivo. M‐MDSCs recruitment is accompanied by sustained immune suppression. Furthermore, we have found that M‐MDSCs impeded T cell proliferation and produced high levels of ROS and Arginase, which can be enhanced by CCL2. Mechanistically, CCL2 stimulated M‐MDSCs led to a significant upregulation of genes, a large part of which accumulated in the mitogen‐activated protein kinase signaling pathway. Treatment of aloesin, MAPK signaling inhibitor, relieved the associated immunosuppressive phenotype induced by CCL2.

**Conclusions:**

Our study indicates that PDAC cells produced CCL2, which promoted localized M‐MDSC recruitment and immune suppression, thereby promoting tumor progression.

## Highlights

CCL2 is significantly upregulated in colon adenocarcinoma

CCL2 influences M‐MDSC recruitment and functionality in colon adenocarcinoma

CCL2 stimulates immune‐suppressive functions of M‐MDSC by activating MAPK signaling

## Graphical abstract

Our data indicate that CCL2 performs a significant role in PDAC progression by recruiting and activating M‐MDSCs. Targeting MDSCs is able to improve antitumoral immunological responses providing with potential applicability of immune‐based combination therapies against an extensive spectrum of solid tumors. These miscellaneous approaches might prove available for tumor therapeutics against solid carcinomas in which M‐MDSCs perform a major role in immune evasion of tumors. These results are promising and need further assessment of the M‐MDSCs‐targeting combination or vaccination approaches for the whole therapeutic capacity of these tactics in PDAC and other carcinomas

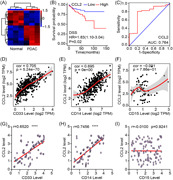

## 1 INTRODUCTION

Systematic immunity is a complicated and multi‐faceted protagonist in malignant disease, influencing the disease from tumorigenesis to outcome in all respects. Immune cells are able to be eliminators of initiation as well as progression in tumors, or stimulators of metastasis, proliferation, infiltration, and drug resistance.^1^ Included in the immune microenvironment of tumors, a variety of cells has been depicted in all tumors with the sophisticated constitution of immune cells depending on the tumor deprivation, distribution, and individual feature of the exact patient. Both innate members (natural killer [NK] cells, myeloid‐derived suppressor cells, dendritic cells [DCs], neutrophils, mast cells, and macrophages) and adaptive members (T lymphocytes and B lymphocytes) exist and crosstalk with the tumor via cytokine and chemokine signaling or through direct contact of superficial receptors, which model the demeanor of the malignancy and its response to treatment.^2^ Incremental decoding of the microenvironment in tumors has contributed to an exponential explosion of the establishment of novel immune‐based biomarkers and the advancement of new drugs that target‐specific pathways for immune therapy.^3^ Previous studies demonstrated that the immunologic milieu of pancreatic ductal adenocarcinoma (PDAC) and the immune profile were unique and need to be deeply explored.^4^

Chemokines are critical modulators of cell migration and cell‐cell crosstalk, and thus have a great impact on malignant development.^5^ Tumor‐related host cells and malignant cells secrete a series of different chemokines, leading to the accumulation and activation of disparate cell types that influence the equilibrium between pro‐tumor and antitumor responses.^6^ It was worth noticing that chemokine (C‐C motif) ligand 2 (CCL2) as the prominent chemokine was eminently overexpressed in digestive system neoplasms.^7^ In the malignant microenvironment, CCL2 crosstalk with C‐C motif chemokine receptor 2 (CCR2) to modulate chemotaxis of monocytes, notably tumor‐associated monocytes, which consequently lead to the modeling of the immune microenvironment and promote cancer progression.^7^ Although M‐MDSCs have been recommended as a potential therapeutic target to treat some sorts of cancers, understanding of the details of its activation and mechanism of tumor progression is limited.

Myeloid‐derived suppressor cells (MDSCs) belong to the cell cluster of heterogeneity, which impedes T‐lymphocyte mediated immune responses against multiple types of tumor, comprising breast, lung, melanoma, and colon carcinomas.^8^ More currently, MDSCs have been verified as mediating immune responses to viral infections, comprised of murine LP‐BM5 retrovirus, simian immunodeficiency virus (SIV), human immunodeficiency virus (HIV), hepatitis C virus (HCV), and cytomegalovirus (CMV).^9^ In mice models, MDSCs are canonically defined as CD11b^+^GR‐1^+^, further subdividing into twin subsets: CD11b^+^Ly6G^+/Hi^Ly6C^±/Lo^ granulocytic MDSCs [G‐MDSCs or polymorphonuclear (PMN)‐MDSCs] and CD11b^+^Ly6G^±/Lo^Ly6C^+/Hi^ monocytic MDSCs (M‐MDSCs).^10^ MDSCs present various co‐expressions of other cellular surface markers more than CD115, IL‐4Rα (CD124), FcγRIII/II (CD16/32), F4/80, and/or TLR‐4. Murine MDSCs might also express disparate combinations of chemokine receptors, notably CX3CR1, CXCR2, CXCR4, and/or CCR2, which are critical for migration of MDSCs from the bone marrow and/or recruitment to tumor niche or sites of infectious disease.^11^ MDSCs impede T‐lymphocyte responses by expression of reactive oxygen species (ROS), arginase‐1 (Arg‐1), nitric oxide (NO), and other mechanisms. To note, M‐MDSCs secrete lower levels of ROS and higher levels of NO, while G‐MDSCs express higher levels of ROS and lower levels of NO, however, both subsets are able to produce some Arg1.^12^, ^13^

In this study, we explored the immune characteristics of PDAC and evaluated the contribution of CCL2 on immune suppression. Our results demonstrated that there was a close relationship between CCL2 and M‐MDSCs in PDAC tissues, which promoted tumor progression. Our data provided evidence for the advancement of agents that target M‐MDSC for immune‐based combination therapy in PDAC.

## 2 MATERIALS AND METHODS

### 2.1 Patients and samples

A total of 100 patients with PDAC were included between January 2017 and December 2019 who underwent surgery for laparoscopic pancreatectomy at the Shanghai General Hospital. The characteristics of cancerous tissues as well as adjacent normal tissues were confirmed by clinical pathology. This study followed the World Medical Association Declaration of Helsinki recommendations and was approved by the Institutional Review Board (IRB) of the Shanghai General Hospital (2017S186).

### 2.2 Quantitative reverse‐transcription PCR (qRT‐PCR)

Total RNA was prepared from target cells utilizing the TRIzol reagent kit (12183555; Life Technologies) and subsequently subjected to reverse transcription. PCR was applied utilizing SYBR® qRT‐PCR Kits (A42352; Clontech Laboratories) and the Step One Plus thermal cycler machine (4376600; Applied Biosystems) in triplicate. β‐Actin and GAPDH were utilized as the internal control. The following primers were utilized for qRT‐PCR: CCL2, forward 5′‐ATGGACCATCCAAGCAGACG‐3′ and reverse 5′‐CCCTTGCTCCACAAGGAAGA‐3′; β‐actin, forward 5′‐CTGGAACGGTGAAGGTGAC‐3′ and reverse 5′‐AAGGGACTTCCTGTAACAATGCA‐3′; CD14, forward 5′‐CATCGTCCAGCTCACAAGGT‐3′ and reverse 5′‐CAGAACCCTAGATGCCCTGC‐3′; CD15, forward 5′‐GCTGCTGATGGGCATCATTG‐3′ and reverse 5′‐CCTGTGGCAGATGGGGAAAT‐3′.

### 2.3 Flow cytometry analysis

All relevant cells were harvested and rinsed utilizing FACS buffer (0.05% sodium azide and 0.5% bovine serum albumin in phosphate‐buffered saline (PBS; Thermo Fisher Scientific). For excluding dead cells, cells were firstly stained with zombie aqua reagent (#423101; Biolegend) in PBS for 10 min at 20°C in the dark. Relevant cells were stained utilizing the APC conjugated anti‐CD14 antibody (#367107; Biolegend), fluorescein isothiocyanate‐conjugated CD33 antibody (#303304; Biolegend), APC conjugated anti‐Gr‐1 antibody (#108424; Biolegend), Brilliant Violet 421 conjugated anti‐Ly6G antibody (#127628; Biolegend), Alexa Fluor® 488 conjugated anti‐CD8 antibody (#100723; Biolegend), APC‐conjugated CD4 antibody (#100412; Biolegend) in FACS buffer as mentioned for at least 30 min at 4°C in the dark. MFI of ROS was detected using CellROX™ Green Flow Cytometry Assay Kit (C10492; Thermo Fisher Scientific). Flow cytometry was applied on the LSRFortessa X‐20 (BD Biosciences). All data were analyzed utilizing FlowJo v10.1 (Treestar).

### 2.4 coculture assay

Transwell coculture experiments were applied in 24‐well plates (Corning Costar). Peripheral blood mononuclear cells (PBMCs) (5 × 10^4^) were seeded as well as cultured in the up wells of 24‐well. Medium with CCL2 and/or anti‐CCL2 antibody was put in down wells. After 24 h coculturing, cells in down wells were collected and analyzed by flow cytometry.

### 2.5 Immunosuppression assay

Freshly CD8^+^ T cells and CD4^+^ T cells were isolated from PBMCs using anti‐CD8 microbeads or anti‐CD4 microbeads (Miltenyi Biotec) and cultured in 96‐well plates with medium containing 0.5 μg/ml of soluble or immobilized anti‐CD3 (05‐50133; American Research Products) and 0.5 μg/ml soluble anti‐CD28 (24‐940‐MSM1, American Research Products). The proliferation of CD8^+^ T cells and CD4^+^ T cells were assessed with a CFSE Cell Division Tracker Kit (BioLegend) after stimulation for 48 h. M‐MDSCs were added at ratios (CD8^+^ T cells to M‐MDSCs = 4:1 and CD4^+^ T cells to M‐MDSCs = 4:1) to CD8^+^ T cells and CD4^+^ T cells for 72 h. Finally, flow cytometry collected CD8^+^ T cells and CD4^+^ T cells with APC‐CD8^+^ T cell antibody (Biolegend) or PE‐CD4^+^ T cells antibody (Biolegend) and then analyzed proliferation rates according to fluorescence attenuation. As for the immunosuppressive effector, ARG1 and iNOS expression in M‐MDSCs were evaluated by western blot analysis, enzyme‐linked immunosorbent assay (ELISA), and flow cytometry.

### 2.6 Animal

Male C57BL/6 mice, weighing approximately 25 g and 8‐week old, were provided by the Shanghai Model Organisms Center, Inc. All experiments were conducted for the sake of minimizing the discomfort and pain of the mice. Mice were maintained in cages with a maximum of five, with free access to food as well as water, and in the base of wood shavings, stationary temperature by an air conditioner, in a 12h light–dark cycle. Intragastric gavage administration was carefully applied, with the animal immobilized, utilizing gavage needle suit to mice. All in vivo procedures were applied according to the Ethical Principles in Animal Experimentation adopted by the Shanghai General Hospital. The Ethics Committee on Animal Use of the Shanghai General Hospital approved the project.

### 2.7 ELISA

The relevant protein level in cell‐free media was estimated using Human Arg ELISA Kit (ab136937; Abcam) according to the manufacturer's protocol.

### 2.8 Western blot analysis

Total proteins inside cells were extracted utilizing lysis buffer (38733; Sigma‐Aldrich). A total of 30–50 μg protein was separated utilizing 10% sodium dodecyl sulfate‐polyacrylamide gel electrophoresis and electronically transferred to polyvinylidene difluoride membranes (IPFL00010; Millipore). Subsequently, immunoblotting was applied utilizing rabbit monoclonal antibodies against MEK2 (ab32517), p‐MEK2 (ab278564), ERK (ab279084), p‐ERK (ab192591), and GAPDH (ab8245'; Abcam). The blots were then visualized utilizing a chemiluminescence detection system (Amersham Pharmacia Biotech).

### 2.9 The Cancer Genome Atlas (TCGA) data analyses

The PDAC data of TCGA were obtained from the UCSC Cancer Browser on the website—https://genome-cancer.ucsc.edu, including normal tissues (*N*  =  41) and primary tumor (*N*  =  286) datasets, and the relevant genome analysis was applied.

### 2.10 Statistical analyses

The results are presented as the means ± *SD*. Comparisons between two sets were analyzed by the Student *t* tests. Statistical analyses were performed by GraphPad Prism 7.0 (GraphPad Software) for both experimental analyses and clinical analyses. For more than two sets of samples, an analysis of variance test was used for statistical analyses. The relationship between G‐CSF and METTL3 levels was determined using Pearson correlation analysis.

## 3 RESULTS

### 3.1 CCL2 increased in PDAC tissues and recruited immature monocytes

**Figure 1:**
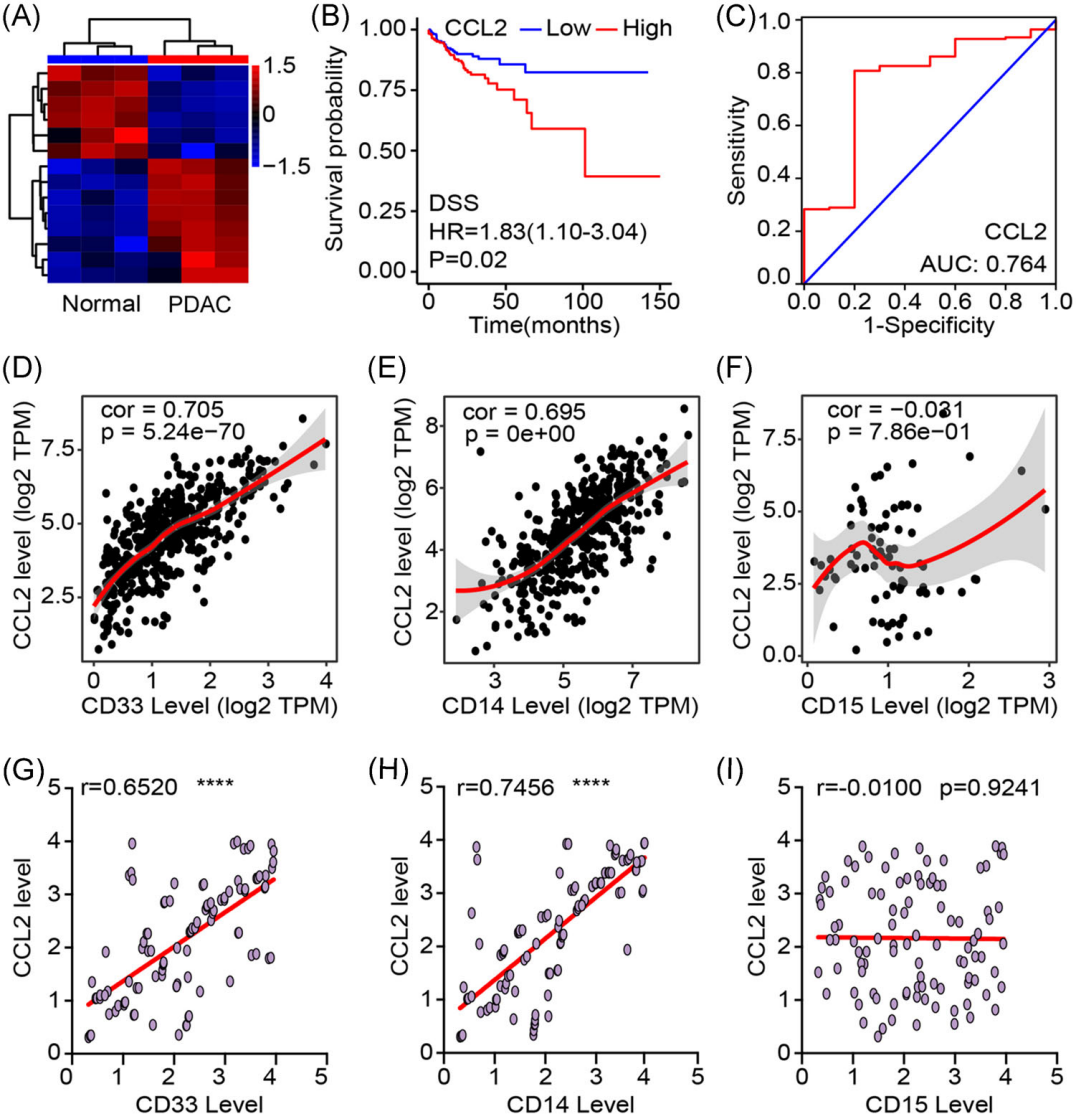
CCL2 increased in PDAC tissues and recruited immature monocytes. (A) Differential genes between normal colon tissues and PDAC tissues were detected by RNA sequencing. (B) DSS of PDAC patients with low or high CCL2 levels. (C) ROC curve indicated that CCL2 can effectively predicate the survival time of PDAC patients. (D–F) Correlation between CD33, CD14, and CD15 with CCL2 in PDAC tissues according to TCGA database. (G–I) In clinical samples, the correlation between CD33, CD14, and CD15 with CCL2. Mean ± *SEM*. CCL2, C‐C motif chemokine ligand 2; DSS, dextran sodium sulfate; PDAC, pancreatic ductal adenocarcinoma; ROC, receiver operator characteristic; TCGA, The Cancer Genome Atlas. ****p < .001

To explore the differences between normal pancreas tissue and PDAC tissues, RNA sequencing was used to test the differential genes. The results showed that several genes were upregulated in PDAC tissues, which included CCL2, a small cytokine that belongs to the CC chemokine family. According to the TCGA database, PDAC patients with high CCL2 expression levels have worse dextran sodium sulfate (DSS) (Figure 1B). CCL2 also effectively indicated a worse prognosis of PDAC patients (Figure 1C). CCL2 recruits monocytes to the sites of the immune microenvironment produced by cancer tissues. To explore the immune recruitment function of CCL2 in PDAC tissues, we analyzed the correlation between CCL2 and immune cells marker. As shown, CCL2 was closely related to immature cells marker, CD33 (Figure 1D). Further investigation showed that monocyte marker, CD14, was in a positive correlation with CCL2 but CD15 not (Figure 1E,F). To further confirm this result, we collected 50 clinical samples, and the results were consistent with bioinformatics prediction. Both CD33 and CD14 were positive with CCL2 except CD15 (Figure 1G–I). Taken together, CCL2 abnormality increased in PDAC tissues and was associated with a poor prognosis. The underlying cause of this phenomenon is that CCL2 may recruit immature monocyte cells into PDAC tissues.

### 3.2 CCL2 recruited M‐MDSCs into PDAC immune microenvironment

**Figure 2:**
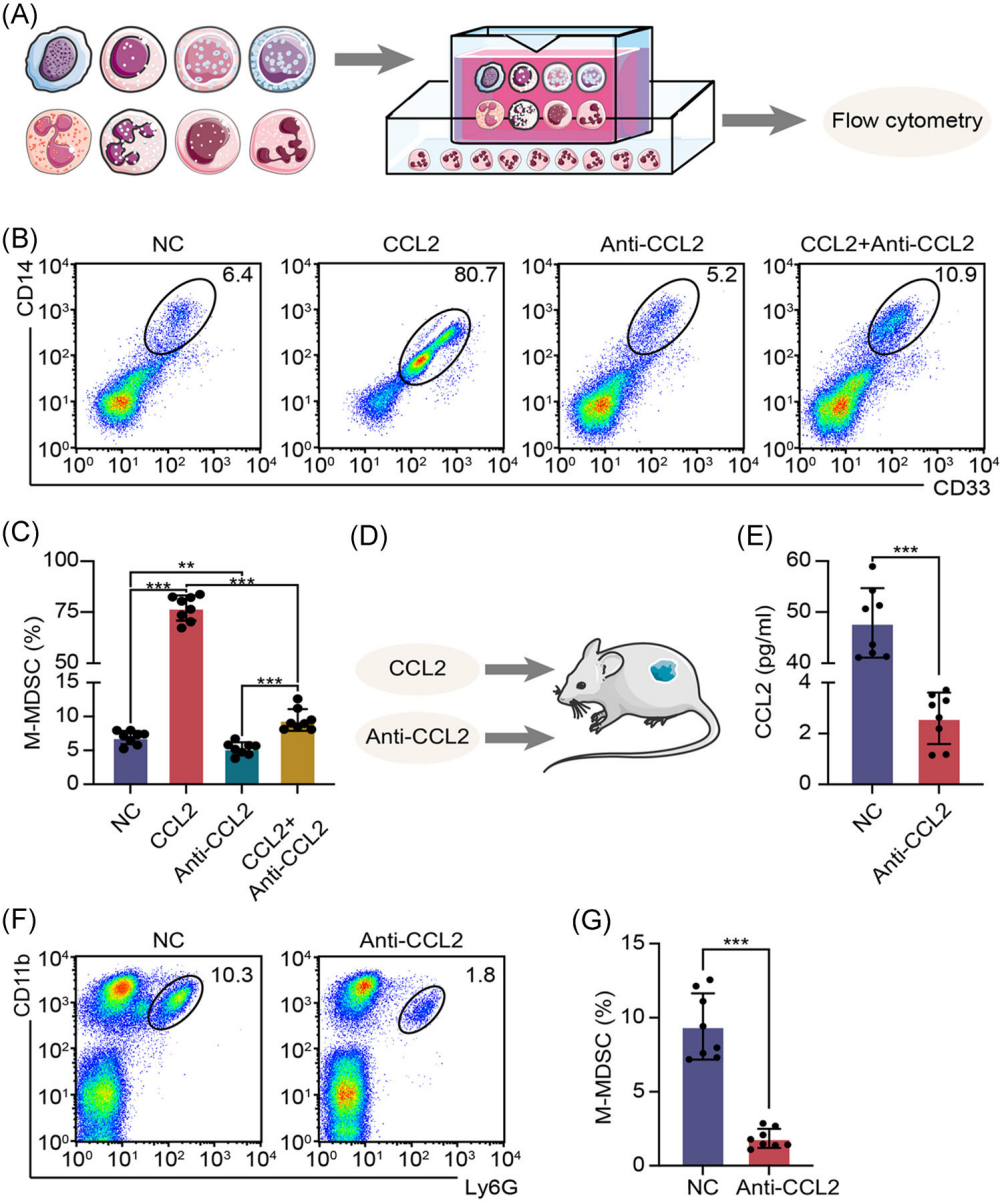
CCL2 recruited G‐MDSCs into PDAC immune environment. (A) Experimental flowchart of immune cells chemotaxis test. (B, C) Images and statistical results of flow cytometry assays to detect M‐MDSCs recruitment. (D) Schematic diagram of experiments in vivo. (E) CCL2 level was tested by ELISA assay. (F, G) Images and statistical results of flow cytometry assays to detect M‐MDSCs proportion in PDAC tissues. Mean ± *SEM*. CCL2, C‐C motif chemokine ligand 2; ELISA, enzyme‐linked immunosorbent assay; G‐MDSC, granulocytic myeloid‐derived suppressor cell; M‐MDSC, monocytic myeloid‐derived suppressor cell; NC, negative control; PDAC, pancreatic ductal adenocarcinoma. **p < .01, ***p < .005

We put human PBMCs into an up petri dish and specific culture medium into a down petri dish to construct a chemotaxis test system and tested the immune cells recruitment function of CCL2 (Figure 2A). To accurately analyze M‐MDSCs in humans and mice, we have developed a flow cytometry gating strategy (Figure S1A,B). Flow cytometry assays and statistical results showed that CCL2 enhanced the chemotaxis of M‐MDSCs, which could be blocked by anti‐CCL2 antibodies (Figure 2B,C). Previous experiments already demonstrated CCL2 was increased in PDAC tissues, thus we treated the PDAC mice model with an anti‐CCL2 antibody for 3 days to block the bio‐function of CCL2, and then PDAC tissues were collected for further experiments (Figure 2D). ELISA assays confirmed that the anti‐CCL2 antibody effectively decreased CCL2 level in PDAC tissues (Figure 2E). Flow cytometry assays and statistical results showed that anti‐CCL2 antibodies effectively restricted M‐MDSCs recruitment in PDAC tissues (Figure 2F,G). These results indicated that CCL2 is a critical chemokine for M‐MDSCs recruitment in PDAC tissues.

### 3.3 CCL2 promoted PDAC progression by enhanced immune suppression ability of M‐MDSCs

**Figure 3:**
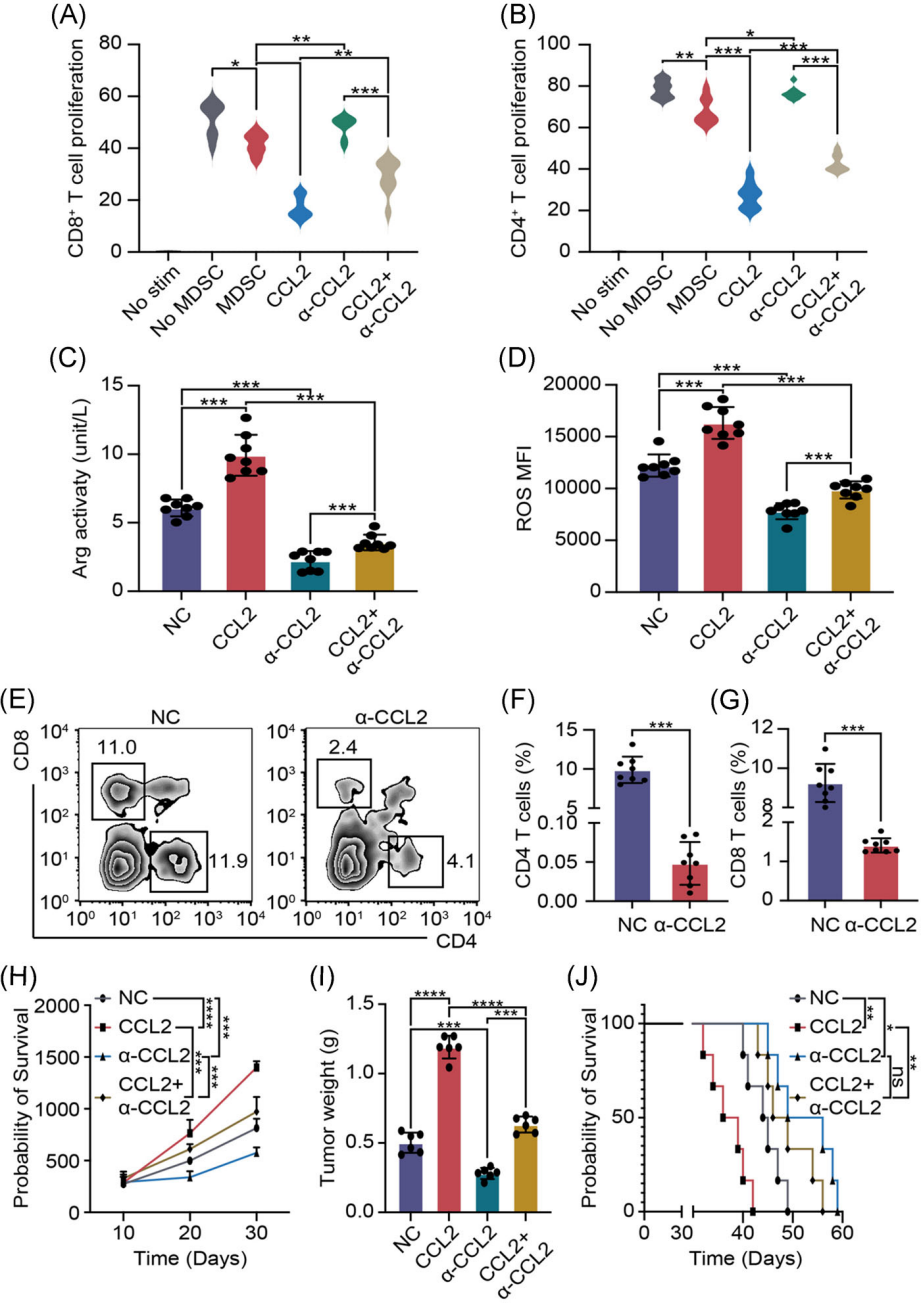
CCL2 promoted PDAC progression by enhanced immune suppression ability of M‐MDSCs. (A, B) Proliferating rates of CD8^+^ T cells and CD4^+^ T cells. (C) ELISA assay was used to test Arg activity. (D) Flow cytometry was used to test ROS level. (E–G) Flow cytometry images and statistical results of CD8^+^ T cells and CD4^+^ T cells proportion. (H) PDAC volume was recorded every 10 days. (I) Tumor weight was recorded after mice were sacrificed. (J) Survival time of PDAC mice models. Mean ± *SEM*. Arg, arginase; CCL2, C‐C motif chemokine ligand 2; ELISA, enzyme‐linked immunosorbent assay; M‐MDSC, monocytic myeloid‐derived suppressor cell; NC, negative control; PDAC, pancreatic ductal adenocarcinoma; ROS, reactive oxygen species. *p < .05, **p < .01, ***p < .005

CCL2 has the function of immune regulation. To explore the effect of CCL2 on M‐MDSCs, coculture system was constructed, in which M‐MDSCs were cocultured with CFSE labeled CD8^+^ T lymphocytes or CFSE labeled CD4^+^ T lymphocytes at a ratio of 1:4 for 48 h. Proliferating rate of CD8^+^ T cells and CD4^+^ T cells were detected by flow cytometry and results showed that CCL2 promoted immune suppression ability of M‐MDSCs, which was blocked by anti‐CCL2 antibody (Figure 3A,B). To confirm these results, we further tested the immunosuppressive effector of M‐MDSCs. ELISA assay and flow cytometry showed that Arg activity and MFI of ROS were increased with CCL2 treatment and anti‐CCL2 antibody partly reversed this phenomenon (Figure 3C,D). We also tested Arg and iNOS by western blot analysis (Figure S2A,B). These results indicated that CCL2 promoted immune suppression ability. In vivo, we treated the PDAC model with CCL2 and/or anti‐CCL2 antibodies. Flow cytometry assays and statistical results showed that CCL2 treatment decreased CD8^+^ T cells and CD4^+^ T cells proportion in PDAC tissues, while anti‐CCL2 antibody inhibited immune suppression ability of CCL2 (Figure 3E–G). CCL2 and/or anti‐CCL2 antibody‐treated mice model and tumor growth rates showed that tumor volume and weight of CCL2 treated group increased faster and anti‐CCL2 antibody inhibited tumor progression (Figure 3H,I). Survival time of the mice model showed that the CCL2 treated group had a worse prognosis and anti‐CCL2 antibody prolonged survival time (Figure 3J). These results showed that CCL2 promoted PDAC progression by enhanced immune suppression ability of M‐MDSCS.

### 3.4 CCL2 promoted immune suppression ability of M‐MDSCs through activating the mitogen‐activated protein kinase (MAPK) signaling pathway

**Figure 4:**
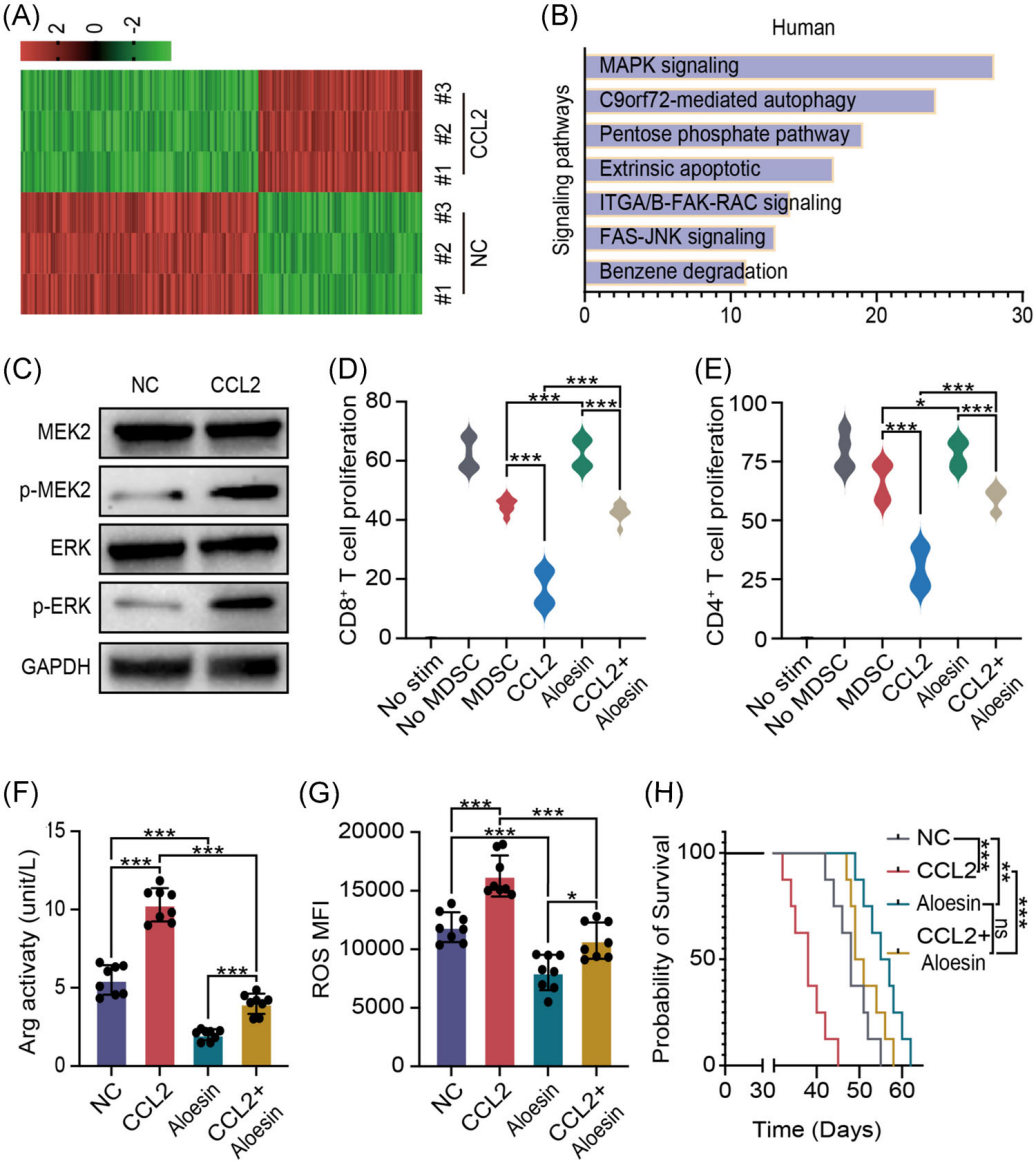
CCL2 promoted the immune suppression ability of M‐MDSCs through activating the MAPK signaling pathway. (A) Hot map of differential genes between PDAC tissues with or without CCL2 treated by RNA sequencing. (B) Differential gene cluster analysis according to sequencing result. (C) Western blot assay was used to test phosphorylation of MEK2 and ERK. (D, E) Proliferating rates of CD8^+^ T cells and CD4^+^ T cells after CCL2 and/or aloesin treated. (F, G) Arg activity and ROS level were detected by ELISA assay and flow cytometry after CCL2 and/or aloesin treatment. (H) Survival time of mice model CCL2 and/or aloesin treatment. Mean ± *SEM*. Arg, arginase; CCL2, C‐C motif chemokine ligand 2; ELISA, enzyme‐linked immunosorbent assay; ERK, endoplasmic reticulum kinase; MAPK, mitogen‐activated protein kinase; M‐MDSC, monocytic myeloid‐derived suppressor cell; NC, negative control; PDAC, pancreatic ductal adenocarcinoma; ROS, reactive oxygen species. *p < .05, **p < .01, ***p < .005

To explore the specific mechanism of CCL2 promoted immune suppression ability of M‐MDSCs, we detected differential genes between normal PDAC tissues and CCL2 treated PDAC tissues (Figure 4A). Cluster analysis showed that differential genes were accumulated in several signaling pathways, especially in the MAPK signaling pathway (Figure 4B). Western blot analysis was used to detected iconic protein of MAPK signaling pathway and quantification of phosphorylation was calculated (Figure S3A,B). As shown, phosphorylation of MEK2 and ERK was increased with CCL2 treatment (Figure 4C). As for immune suppression ability, CCL2 and/or aloesin, the effective MAPK signaling inhibitor, were treated M‐MDSCs for 48 h and then cocultured with CD8^+^ T cells or CD4^+^ T cells. Proliferating rates of CD8^+^ T cells and CD4^+^ T cells showed that inhibiting MAPK signaling pathway can effectively blocking immune suppression enhanced function of CCL2 (Figure 4D,E). Furthermore, we also tested Arg activity and ROS level by ELISA assay and flow cytometry. As shown, aloesin partly reversed the immune regulatory function of CCL2 (Figure 4F,G). Survival time of PDAC mice also showed that aloesin treatment improve the prognosis of CCL2 treated mice (Figure 4H). These results showed that CCL2 promoted the immune suppression ability of M‐MDSCs through activating the MAPK signaling pathway. Taken together, CCL2 produced by PDAC cells is essential for the recruitment and activation of M‐MDSCs for immunosuppression.

## 4 DISCUSSION

The immunological microenvironment in malignant neoplasms contributes to tumor growth and metastasis, and the tumor‐infiltrating immune cells affect patients’ not only prognosis but overall survival.^14^ Although plenty of immune cells, stroma cells, and immunomodulatory factors have been associated with soaring tumor metastatic potential, discovering immune checkpoints (ICP) at the earliest phase of tumor advancement and influencing such discoveries to keep tumors from advancement and progression has become an unprecedented challenge notably in PDAC.^15^ Hereby, centering on CCL2 in PDAC, we demonstrated that CCL2 manifests as a pro‐tumoral factor that modulates M‐MDSC recruitment and functionality and rears a tumor‐tolerant immune microenvironment. We also showed that CCL2 enhanced immune suppression ability and promoted PDAC progression.^16^ Antibody‐associated antigen neutralization of tumorigenic CCL2 suspends cancer development, giving a golden chance for the potent therapeutics of CCL2 blockades and neutralization antibodies in PDAC treatment and prevention.^17^

MDSCs have been identified as the crucial member of the tumor immune microenvironment of a lot of many carcinomas, and our cognition of the influencing factors that modulate MDSCs migration, accumulation, and functionality keeps expanding.^18^ For instance, granulocyte stimulating factor recruits the immature myeloid‐derived cells that enriched in PDAC in experimental mice, elicited CD8^+^ T lymphocyte‐mediated antitumor adaptive immunity, and regulated MDSC propagation and functionality in the spleen.^16^ In the model of inflammation‐associated CRC, established by dextran sodium sulfate (DSS)–azoxymethane (AOM), CXCR2 knockout contributed to decremental tumor number, and MDSC‐derived CXCR2 was a critical potential mechanism.^16^ According to our results, CCL2 leads to infiltrating M‐MDSC recruitment and additionally affects MDSC‐regulated impedance of CD4^+^ T and CD8^+^ T lymphocytes. A few factors have been involved in the T‐lymphocyte‐suppressive functions of MDSCs.

Our results upheld that CCL2 affects ROS generation and Arg1 expression. Although MDSC‐generated ROS is proved to affect TCR ζ chain expression, our analysis provided the crucial components of the signaling pathway by which CCL2 modulates ROS generation in M‐MDSCs.^19^ ROS performs a plethora of roles in tumorigenesis and imposes influences on not merely tumor demise and survival yet responses to radiation therapy and chemotherapy. Our results surrounding ROS were specifically centered on how CCL2 modulates ROS production in M‐MDSCs, and subsequently, its role played in impeding T lymphocytes. We discovered that CCL2 could also affect Arg1 expression inside M‐MDSCs. The TCR complex undergoing tyrosine nitrosylation is another critical mechanism where MDSCs regulate impedance of T lymphocytes, and our results upheld that CCL2 performs a role in this territory. MDSCs use some overlapping mechanisms of immunosuppression and previous studies have often paid close heed to block selective MDSC functions. Contrariwise, our work, which used various mice models, uncloaked that CCL2 modulates a few of suppressive functionalities of MDSCs with contributions to both the adaptive and innate immunity to PDAC carcinogenesis. What is more, we elucidated that CCL2 stimulated MAPKs signaling pathway, which was crucial in immunosuppression modulation.

Hitherto, immune therapeutics were generally classified into three specific categories: ICP inhibitors, adoptive cell therapies, and cancer vaccines. In recent years, ICP blockades have manifested stupendous prospects in bladder cancer, non‐small‐cell lung cancer, renal cell carcinoma, and melanoma.^20^, ^21^ Specifically, agents targeting the CTLA4, PD‐L1, and PD‐1 checkpoints present extremely promising for these solid carcinomas. Nevertheless, it still needs to be explored the reason why exactly targeting these ICP inhibitors is more efficacious in some solid malignant diseases than others and is a part, but not all, of sufferers bearing the same carcinomas.^22^, ^23^ Our discoveries illustrated that CCL2 affects the activation of MAPKs in M‐MDSCs and that CCL2 stimulated M‐MDSC‐modulated impedance of T lymphocytes raising the question of whether M‐MDSCs, CCL2, and M‐MDSC/CCL2‐regulated suppression of T lymphocytes in tumor microenvironment might be significant factors to consider when identifying the reason why current ICP therapies might not take effect on all tumor‐bearing patients. We consider that serum or intratumoral M‐MDSC and CCL2 levels might serve as useful factors to select responsive patients for current ICP therapies and that neutralization antibodies of CCL2 might be necessary to augment responses to current ICP targeted therapies in some selected patients. Future research needs to be implemented in human PDAC for better characterizing other subsets and functions in MDSCs.

Taken together, our data indicate that CCL2 performs a significant role in PDAC progression by recruiting and activating M‐MDSCs. Targeting MDSCs is able to improve antitumoral immunological responses providing with potential applicability of immune‐based combination therapies against an extensive spectrum of solid tumors. These miscellaneous approaches might prove available for tumor therapeutics against solid carcinomas in which M‐MDSCs perform a major role in immune evasion of tumors. We look forward that CCL2 targeted antagonists and MDSC targeted drugs can enter the next step of clinical practice and eventually be applied in clinical practice. These results are promising and need further assessment of the M‐MDSCs‐targeting combination or vaccination approaches for the whole therapeutic capacity of these tactics in PDAC and other carcinomas.

## ROLE OF THE FUNDER/SPONSOR

The funders had no role in the design and conduct of the study; collection, management, analysis, and interpretation of the data; preparation, review, or approval of the manuscript; and decision to submit the manuscript for publication.

## CONFLICT OF INTERESTS

We declare that we do not have any commercial or associative interest that represents a conflict of interest in connection with the work submitted.

## AUTHOR CONTRIBUTIONS

Haitao Gu and Feng Sun designed research; Haitao Gu, Wensheng Deng, and Ke Wu performed experiments; Haitao Gu, Feng Sun, and Zhong Zheng recruited patients; Haitao Gu, Wensheng Deng, Ke Wu, and Feng Sun analyzed data and wrote the manuscript. All authors read and approved the manuscript.

## ETHICS APPROVAL AND CONSENT TO PARTICIPATE

For all of the patients who participated in this study, written informed consent was obtained. It was approved by the Institutional Review Board (IRB) of the Shanghai General Hospital according to the Chinese Ethical Regulations. All animal experiments complied with the Ethics Committee on Animal Use of the Shanghai General Hospital.

## Supporting information

Supplementary information.

Supplementary information.

Supplementary information.

Supplementary information.

## Data Availability

The detailed procedures of methods, four figures are attached. All data, models, and code generated or used during the study appear in the submitted article.
