# Supplementary figures and images for: CCL2 produced by pancreatic ductal adenocarcinoma is essential for the accumulation and activation of monocytic myeloid‐derived suppressor cells
Source: Immun Inflamm Dis. 2021 Sep 15;9(4):1686–95. doi: 10.1002/iid3.523 (PMC8589368; doi:10.1002/iid3.523)

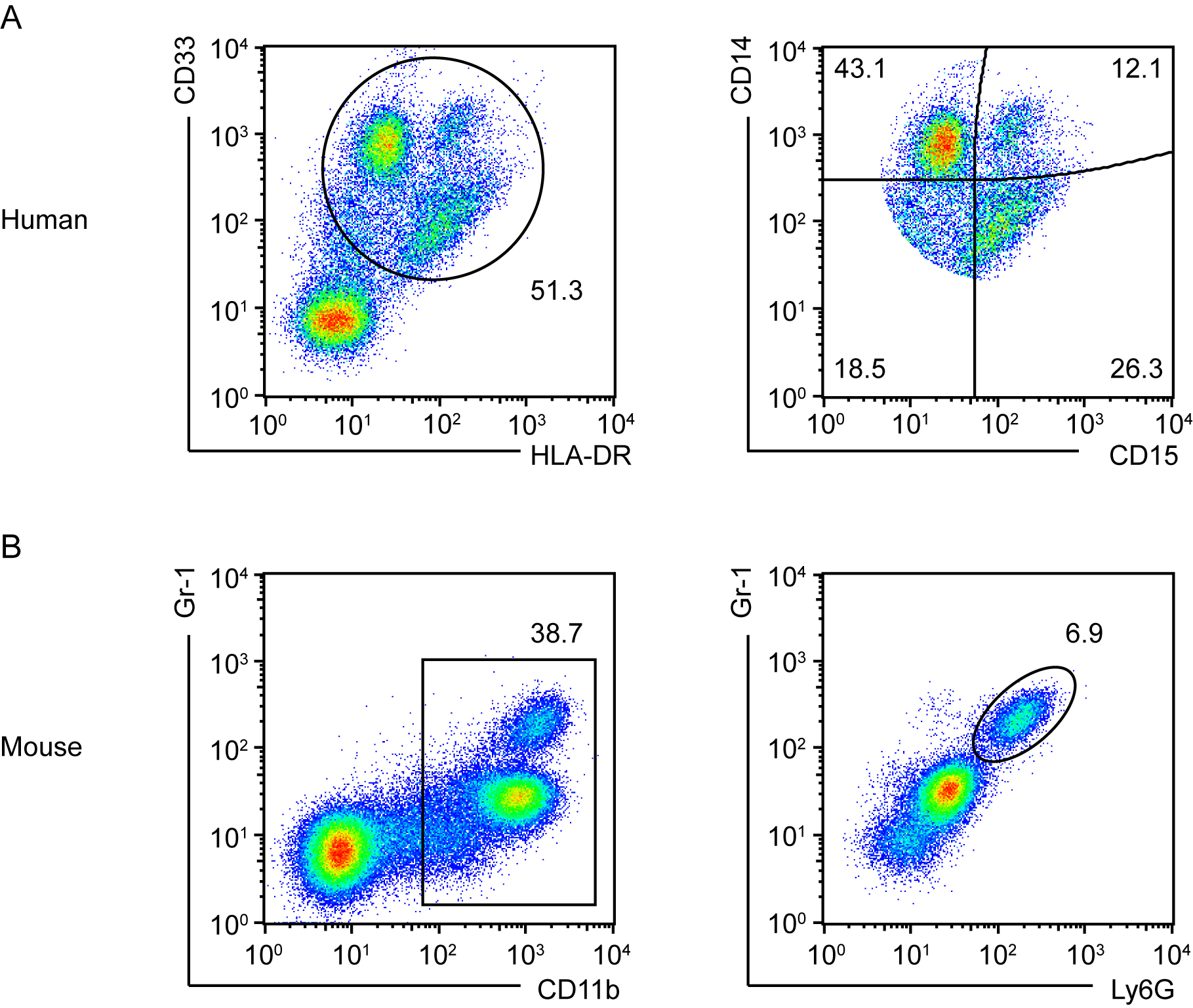

Supplement: Supplementary file 1 — Supplementary information. [file IID3-9-1686-s003.jpg]

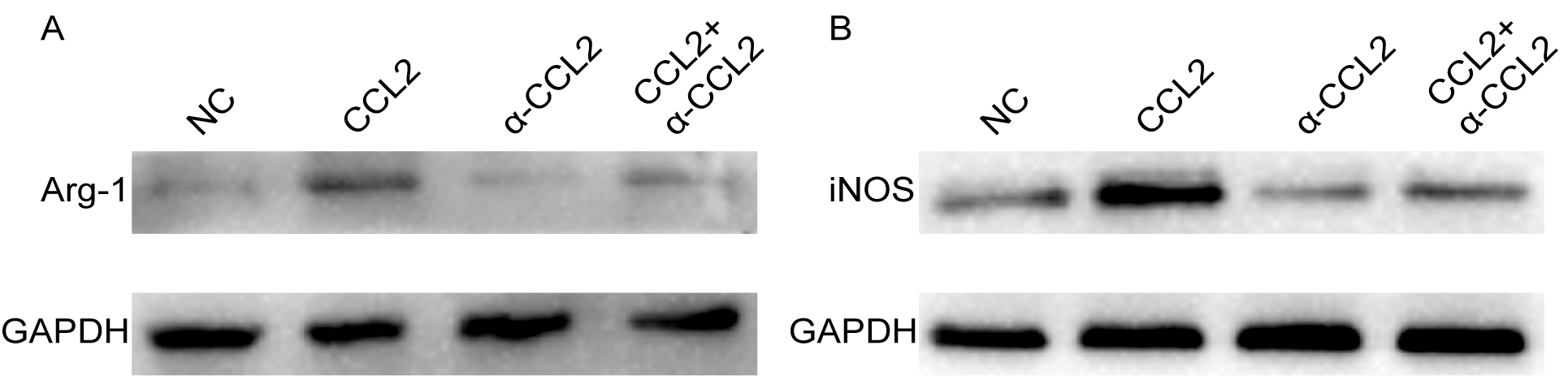

Supplement: Supplementary file 2 — Supplementary information. [file IID3-9-1686-s004.jpg]

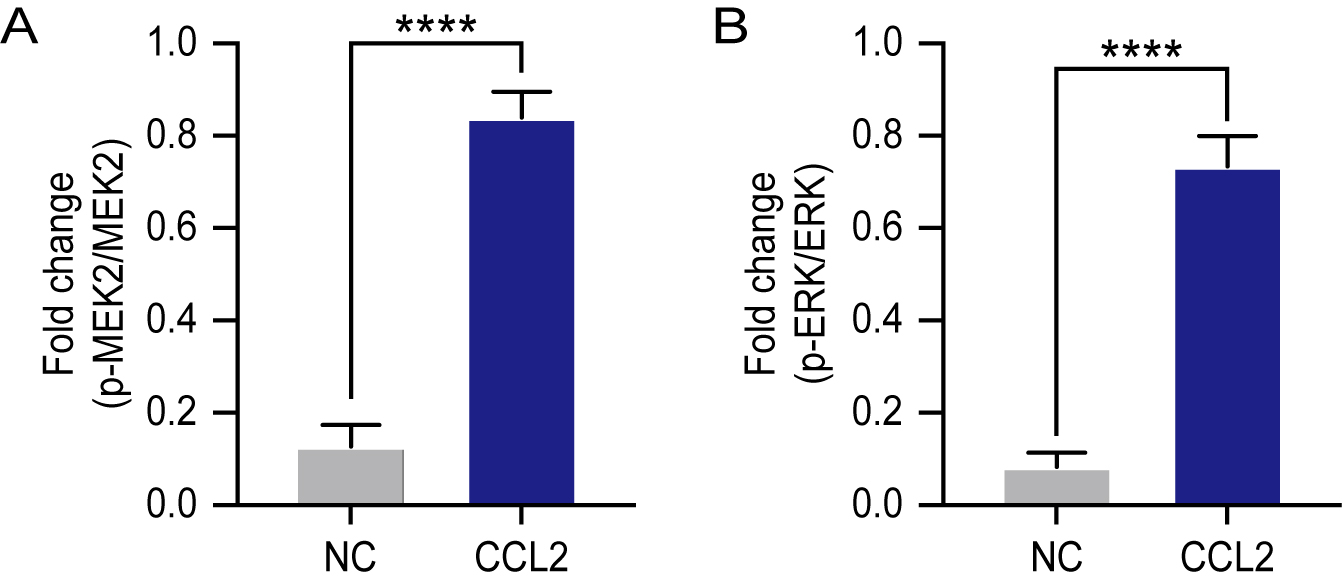

Supplement: Supplementary file 3 — Supplementary information. [file IID3-9-1686-s001.jpg]
