# Supplementary material for: CCL2 produced by pancreatic ductal adenocarcinoma is essential for the accumulation and activation of monocytic myeloid‐derived suppressor cells
Source: Immun Inflamm Dis. 2021 Sep 15;9(4):1686–95. doi: 10.1002/iid3.523 (PMC8589368; doi:10.1002/iid3.523)
Supplement: Supplementary file 4 — Supplementary information. [file IID3-9-1686-s002.docx]

**Figure S1**Flow cytometry gating strategy of M-MDSCs in human and mice. A. Images of flow cytometry assays to detect M-MDSCs in human. B. Images of flow cytometry assays to detect M-MDSCs in mice.

**Figure S2** CCL2 increased Arg-1 and iNOS in M-MDSCs. A, B. Western blot assay was used to test the protein level of Arg-1 and iNOS in M-MDSCs.

**Figure S3**Western blotting quantification of MEK2 and ERK phosphorylation. A. Quantification of MEK2 phosphorylation. B. Quantification of ERK phosphorylation. Mean▒±▒SEM, ****P▒<▒0.001.
